# Supplementary material for: Structure of the F-tractin–F-actin complex
Source: J Cell Biol. 2025 Feb 10;224(4):e202409192. doi: 10.1083/jcb.202409192 (PMC11809415; doi:10.1083/jcb.202409192)
Supplement: Table S1 — shows list of primers and plasmids used in this study. [file jcb_202409192_tables1.docx]

**Structure of the F-tractin–F-actin complex**

Dmitry Shatskiy, Athul Sivan, Roland Wedlich-Söldner and Alexander Belyy

Supplementary information

**Supplementary Table 1. List of primers and plasmids used in this study.**

| **Plasmid** | **Description** | **Reference** |
| --- | --- | --- |
| pB791 pET28a eAF1521 | Gene, encoding ADP-ribose binding protein AF 1521 (uniprot O28751) with two mutations K35E and Y145R (Kathrin Nowak Nat Commun 2020), was synthesized by Twist Biosciences and cloned into pET28a vector using NdeI/NotI sites. | This study |
| pB1030 pET28a F-tractin | Gene, encoding F-tractin in fusion with mCherry-6xHis, was synthesized by Twist Biosciences and cloned into pET28a vector using NcoI/HindIII. | This study |
| pB1043 pET28a F-tractin_opt_ | Primers catggccccacgacgttcagtcggggagctcagattgctttttgaagcgcgggcagcatctgccgccg and gatccggcggcagatgctgcccgcgcttcaaaaagcaatctgagctccccgactgaacgtcgtggggc were annealed to generate DNA sequence encoding F-tractin_opt_ and inserted into pB1030 digested with NcoI and BamHI. | This study |
| pB1044 pET28a F-tractin_opt_ F29A | Primers catggccccacgacgttcagtcggggagctcagattgcttgcggaagcgcgggcagcatctgccgccg and gatccggcggcagatgctgcccgcgcttccgcaagcaatctgagctccccgactgaacgtcgtggggc were annealed to generate DNA sequence encoding F29A F-tractin_opt_ and inserted into pB1030 digested with NcoI and BamHI. | This study |
| pB1045 pET28a F-tractin_opt_ R19A | Primers catggccccatcacgttcagtcggggagctcagattgctttttgaagcgcgggcagcatctgccgccg and gatccggcggcagatgctgcccgcgcttcaaaaagcaatctgagctccccgactgaacgtgatggggc were annealed to generate DNA sequence encoding R19A F-tractin_opt_ and inserted into pB1030 digested with NcoI and BamHI. | This study |
| pB656 pET28 MBP TcART | His-tagged TcART in MBP fusion. | [1] |
| pB1055 pEGFP-N1 | Vector for expression of EGFP fusion genes in eukaryotic hosts. | Addgene plasmid # 13031 |
| pB1057 pEGFP-N1 F-tractin | Primers tatactcgagatggctcgcccgagaggggctggtccatgcagcccgggtttagagcgggccccacgacgttcagtcggggagctc and tataggatcccctgcggccgctgctgcggctacggctgcgcaccgcgcttcaaaaagcaatctgagctccccgactgaacgtcg were annealed and PCR-amplified to generate DNA sequence encoding F-tractin_,_ cut with XhoI and BamHI and inserted into pB1055 digested with XhoI and BamHI. | This study |
| pB1058 pEGFP-N1 F-tractin_opt_ | Primers tcgagatgGCCCCACGACGTTCAGTCGGGGAgCTcAGATTGCTTTTTGAAGCGCGGGCAGCAtctGCCGCCggg and gatccccGGCGGCagaTGCTGCCCGCGCTTCAAAAAGCAATCTgAGcTCCCCGACTGAACGTCGTGGGGCcatc were annealed to generate DNA sequence encoding F-tractin_opt_ and inserted into pB1055 digested with XhoI and BamHI. | This study |
| pB1059 pEGFP-N1 F-tractin_opt_ F29A | Primers tcgagatgGCCCCACGACGTTCAGTCGGGGAgCTcAGATTGCTTGCGGAAGCGCGGGCAGCAtctGCCGCCggg and gatccccGGCGGCagaTGCTGCCCGCGCTTCCGCAAGCAATCTgAGcTCCCCGACTGAACGTCGTGGGGCcatc were annealed to generate DNA sequence encoding F29A F-tractin_opt_ and inserted into pB1055 digested with XhoI and BamHI. | This study |
| pB1060_pEGFP-N1 F-tractin_opt_ R19A | Primers tcgagatgGCCCCATCACGTTCAGTCGGGGAgCTcAGATTGCTTTTTGAAGCGCGGGCAGCAtctGCCGCCggg and gatccccGGCGGCagaTGCTGCCCGCGCTTCAAAAAGCAATCTgAGcTCCCCGACTGAACGTGATGGGGCcatc were annealed to generate DNA sequence encoding R19A F-tractin_opt_ and inserted into pB1055 digested with XhoI and BamHI. | This study |
| pEGFP-N1 Lifeact | Primers aagcttcgaattcATGGGTGTCGCAGATTTGATCAAGAAATTCGAAAGCATCTCAAAGGAAGAAggg and tcgaattcATGGGTGTCGCAGATTTGATCAAGAAATTCGAAAGCATCTCAAAGGAAGAAggggatc were annealed to generate DNA sequence encoding Lifeact and inserted to pB1055 with BamHI and HindIII | This  study |

**References**

1. Belyy, A., et al. Mechanism of threonine ADP-ribosylation of F-actin by a Tc toxin. *Nat Commun* **13**, 4202 (2022).
