# Supplementary material for: Structure of the F-tractin–F-actin complex
Source: J Cell Biol. 2025 Feb 10;224(4):e202409192. doi: 10.1083/jcb.202409192 (PMC11809415; doi:10.1083/jcb.202409192)
Supplement: Table S2 — shows data points. [file jcb_202409192_tables2.docx]

**Structure of the F-tractin–F-actin complex**

Dmitry Shatskiy, Athul Sivan, Roland Wedlich-Söldner and Alexander Belyy

Supplementary information

**Supplementary Table 2. Data points**

**Figure 2 D**

|  |  | F-actin in the pellet | | |
| --- | --- | --- | --- | --- |
|  | Peptide, µM | Repeat 1 | Repeat 2 | Repeat 3 |
| F-tractin | 0 | 0,036788 | 0,030874 | 0,013011 |
|  | 1 | 0,052087 | 0,032888 | 0,047528 |
|  | 10 | 0,092603 | 0,600815 | 0,323365 |
|  | 100 | 0,999376 | 0,989657 | 0,931572 |
| F-tractin_opt_ | 0 | 0,041618 | 0,054048 | 0,049545 |
|  | 1 | 0,098435 | 0,057915 | 0,103265 |
|  | 100 | 0,099007 | 0,095989 | 0,158954 |
|  | 1000 | 0,238373 | 0,117922 | 0,212762 |
| Lifeact | 0 | 0,041618 | 0,054048 | 0,049545 |
|  | 10 | 0,07218 | 0,09382 | 0,137443 |
|  | 100 | 0,1046 | 0,117135 | 0,14654 |
|  | 1000 | 0,339777 | 0,164721 | 0,309232 |

**Figure 2 G**

|  | Fraction of **F-tractin_opt_** bound | | |
| --- | --- | --- | --- |
| F-actin, µM | Repeat 1 | Repeat 2 | Repeat 3 |
| 2 | 0,113982 | 0,125633 | 0,150215 |
| 5 | 0,211009 | 0,27957 | 0,318841 |
| 10 | 0,480176 | 0,433657 | 0,418605 |
| 20 | 0,569748 | 0,578018 | 0,523474 |
| 40 | 0,557282 | 0,621935 | 0,643478 |

|  | Fraction of F29A **F-tractin_opt_** bound | | |
| --- | --- | --- | --- |
| F-actin, µM | Repeat 1 | Repeat 2 | Repeat 3 |
| 2 | 0,02963 | 0,031317 | 0,036 |
| 5 | 0,020561 | 0,008869 | 0,001393 |
| 10 | 0,051546 | 0,016783 | 0,023887 |
| 20 | 0,035971 | 0,051087 | 0,050633 |
| 40 | 0,071269 | 0,046373 | 0,065217 |

|  | Fraction of R19A **F-tractin_opt_** bound | | |
| --- | --- | --- | --- |
| F-actin, µM | Repeat 1 | Repeat 2 | Repeat 3 |
| 2 | 0,078051 | 0,069519 | 0,125538 |
| 5 | 0,150979 | 0,148265 | 0,193573 |
| 10 | 0,31152 | 0,304965 | 0,249039 |
| 20 | 0,358879 | 0,357945 | 0,31634 |
| 40 | 0,488372 | 0,519149 | 0,323824 |

**Figure 3 C**

| Pearson Correlation coefficient for each cell analyzed | | | | | |
| --- | --- | --- | --- | --- | --- |
| EGFP | Lifeact | WT F- tractin | F-tractin_opt_ | F29A F-tractin_opt_ | R19A F-tractin_opt_ |
| 0.10954 | 0.76643 | 0.78651 | 0.67295 | 0.30638 | 0.52439 |
| 0.17413 | 0.35274 | 0.83482 | 0.4789 | 0.35424 | 0.51659 |
| 0.20843 | 0.89957 | 0.81213 | 0.43512 | 0.05418 | 0.21147 |
| 0.56273 | 0.79351 | 0.73699 | 0.71914 | -0.09177 | 0.45312 |
| 0.25628 |  | 0.81463 | 0.24809 | 0.24243 | 0.58155 |
| -0.11892 | 0.76541 | 0.51708 | 0.17482 | 0.33315 | 0.34979 |
| -0.14581 | 0.75694 | 0.70416 | 0.74647 | -0.1638 | 0.21057 |
| -0.24725 | 0.63211 | 0.425 | 0.81435 | 0.10337 | 0.49853 |
| -0.06922 | 0.6686 | 0.41105 | 0.72387 | 0.49504 | 0.52201 |
| -0.33835 | 0.44503 | 0.6836 | 0.90307 | 0.07811 | -0.00839 |
| -0.35467 | 0.64123 | 0.65265 | 0.51485 | 0.00323 | 0.47492 |
| 0.18496 | 0.2489 | 0.46728 | 0.77871 | -0.05819 | 0.57243 |
| -0.46832 | 0.76761 | 0.86762 | 0.78517 | 0.24261 | 0.41911 |
| -0.02363 | 0.72541 | 0.84982 | 0.30793 | -0.06018 | 0.46935 |
| 0.12068 | 0.6592 | 0.30961 | 0.45293 | 0.1486 | 0.53496 |
| 0.56515 | 0.69321 | 0.67704 | 0.571 | 0.152 | 0.08256 |
| 0.0863 | 0.9123 | 0.68198 | 0.65055 | -0.10862 | 0.48473 |
| 0.45723 | 0.834 | 0.34589 | 0.76628 |  | -0.15254 |
| 0.44998 | 0.87403 | 0.46251 | 0.37407 |  | 0.52933 |
| 0.31887 | 0.70696 | 0.71653 | 0.53954 |  | 0.26181 |
| 0.0651 | 0.73191 | 0.81744 | 0.70429 |  | 0.72892 |
| 0.54355 | 0.4622 | 0.82248 | 0.62755 |  | 0.44463 |
| -0.13263 | 0.21844 | 0.78101 | 0.80792 |  | 0.56268 |
| 0.17658 | 0.78344 | 0.60454 | 0.9354 |  | 0.44615 |
| 0.06247 | 0.89871 | 0.25161 |  |  | 0.24766 |
| 0.2267 | 0.76498 | 0.64922 |  |  | 0.16194 |
| -0.22143 | 0.69642 | 0.44011 |  |  | -0.06174 |
| 0.34798 | 0.85502 | 0.70425 |  |  | 0.01029 |
| 0.22207 | 0.70383 | 0.81156 |  |  | 0.67663 |
| -0.09331 | 0.78924 | 0.63599 |  |  | 0.79555 |
| 0.15295 | 0.17659 | 0.88315 |  |  | 0.4683 |
| 0.17203 | 0.71164 | 0.7852 |  |  |  |
| 0.0401 | 0.93357 | 0.40483 |  |  |  |
| 0.01924 | 0.61379 | 0.73442 |  |  |  |
| -0.43342 | 0.82511 | 0.72687 |  |  |  |
| 0.49162 | 0.64172 | 0.78591 |  |  |  |
| 0.10954 | 0.91147 | 0.28591 |  |  |  |
| 0.17413 | 0.88287 |  |  |  |  |
| 0.20843 |  |  |  |  |  |
| 0.56273 |  |  |  |  |  |
| 0.25628 |  |  |  |  |  |
| -0.11892 |  |  |  |  |  |
| -0.14581 |  |  |  |  |  |
| -0.24725 |  |  |  |  |  |
| -0.06922 |  |  |  |  |  |
| -0.33835 |  |  |  |  |  |
| -0.35467 |  |  |  |  |  |
| 0.18496 |  |  |  |  |  |
| -0.46832 |  |  |  |  |  |
| -0.02363 |  |  |  |  |  |
| 0.12068 |  |  |  |  |  |
| 0.56515 |  |  |  |  |  |
| 0.0863 |  |  |  |  |  |
| 0.45723 |  |  |  |  |  |
| 0.44998 |  |  |  |  |  |
| 0.31887 |  |  |  |  |  |
| 0.0651 |  |  |  |  |  |
| 0.54355 |  |  |  |  |  |
| -0.13263 |  |  |  |  |  |
| 0.17658 |  |  |  |  |  |
| 0.06247 |  |  |  |  |  |
| 0.2267 |  |  |  |  |  |
| -0.22143 |  |  |  |  |  |
| 0.34798 |  |  |  |  |  |
| 0.22207 |  |  |  |  |  |
| -0.09331 |  |  |  |  |  |
| 0.15295 |  |  |  |  |  |
| 0.17203 |  |  |  |  |  |
| 0.0401 |  |  |  |  |  |
| 0.01924 |  |  |  |  |  |
| -0.43342 |  |  |  |  |  |
| 0.49162 |  |  |  |  |  |

**Figure 3 D**

| Signal to noise ratio between fibers and cytosol | | | | | |
| --- | --- | --- | --- | --- | --- |
| EGFP | Lifeact-EGFP | WT-F-tractin | F-tractin_opt_ | R19A F-tractin_opt_ | F29A F-tractin_opt_ |
| 0.93143 | 1.45751 | 1.33348 | 1.47235 | 1.2139 | 0.98474 |
| 0.95471 | 1.35744 | 1.41893 | 1.21246 | 1.13762 | 1.06445 |
| 0.99076 | 2.08166 | 1.58483 | 1.29509 | 1.13194 | 1.00508 |
| 1.43149 | 1.36719 | 1.57929 | 1.43808 | 1.49593 | 0.96149 |
| 1.16078 | 1.82324 | 1.44522 | 1.16886 | 1.51873 | 1.00435 |
| 0.88951 | 1.80568 | 1.22118 | 1.04772 | 1.10119 | 0.94315 |
| 0.9278 | 1.35682 | 1.5103 | 1.39709 | 1.08491 | 0.96876 |
| 0.83267 | 1.40459 | 1.38392 | 1.3969 | 1.24567 | 0.98419 |
| 0.96255 | 1.2232 | 1.11582 | 1.25325 | 1.17949 | 0.99605 |
| 0.88486 | 1.37541 | 1.40246 | 1.95053 | 0.96147 | 0.93947 |
| 0.78978 | 1.13439 | 1.24236 | 1.36565 | 1.16493 | 0.7677 |
| 0.96659 | 1.56602 | 1.35265 | 1.33382 | 1.15306 | 0.8062 |
| 0.82036 | 1.69603 | 1.5102 | 1.85815 | 1.25075 | 0.93819 |
| 0.95933 | 1.41159 | 1.62271 | 1.31485 | 1.2465 | 0.82586 |
| 1.11046 | 1.28347 | 1.06501 | 1.40679 | 1.32674 | 1.0963 |
| 1.68816 | 1.71423 | 1.59317 | 1.20003 | 1.07391 | 0.9292 |
| 1.02078 | 1.56521 | 1.48624 | 1.32228 | 1.25982 | 0.95362 |
| 1.46518 | 1.66781 | 1.30111 | 1.3628 | 0.98338 | 0.87363 |
| 1.14309 | 1.14496 | 1.22929 | 1.00848 | 1.30404 | 1.01816 |
| 1.09081 | 1.4968 | 1.2017 | 1.50009 | 1.04494 | 0.92906 |
| 0.94961 | 1.39773 | 1.50526 | 1.48655 | 1.31922 | 0.98289 |
| 1.27376 | 1.07983 | 1.44636 | 1.18407 | 1.25404 | 0.98317 |
| 0.8864 | 1.94218 | 1.30888 | 1.32242 | 1.36438 | 1.08573 |
| 1.00936 | 1.46837 | 1.43726 | 1.54994 | 1.36986 | 0.87293 |
| 1.25217 | 1.48475 | 1.17538 | 1.23927 | 1.29182 | 1.12053 |
| 0.98153 | 1.18999 | 1.24739 | 1.35246 | 1.06318 | 1.0027 |
| 1.09524 | 1.64394 | 1.12044 | 1.40124 | 1.00148 | 0.88115 |
| 1.1836 | 1.32829 | 1.55151 | 1.39492 | 1.01756 |  |
| 1.32673 | 1.40072 | 1.59863 | 1.50744 | 1.26424 |  |
| 1.05535 | 0.99022 | 1.31439 | 1.60272 | 1.49536 |  |
| 1.00154 | 1.72402 | 1.44544 | 1.24214 | 1.23221 |  |
| 1.1615 | 1.32937 | 1.458 | 1.35622 | 1.1805 |  |
| 0.73829 | 1.06726 | 1.16944 | 1.11505 | 1.4765 |  |
| 0.91474 | 1.78097 | 1.47513 | 1.20096 | 1.49537 |  |
| 0.77519 | 1.29874 | 1.2122 | 1.26758 | 1.07212 |  |
| 1.22382 | 1.72901 | 1.36634 |  |  |  |
| 0.98812 | 1.87339 | 1.0831 |  |  |  |
| 0.94996 | 1.10937 | 1.27878 |  |  |  |
| 0.9604 | 1.27535 | 1.64334 |  |  |  |
| 0.9044 | 1.60983 | 1.23905 |  |  |  |
| 1.15431 | 1.19784 | 1.48975 |  |  |  |
| 1.08726 | 1.77929 | 1.76165 |  |  |  |
| 0.89257 | 1.41789 | 1.73881 |  |  |  |
|  | 1.10905 | 1.17378 |  |  |  |
|  | 1.22208 | 1.39734 |  |  |  |
|  | 1.6521 | 1.40647 |  |  |  |
|  | 1.49378 | 1.1718 |  |  |  |
|  | 1.40108 | 1.25931 |  |  |  |
|  | 1.29041 | 1.18811 |  |  |  |

**Figure 3 E**

| Nuclear signal enrichment | | | | | |
| --- | --- | --- | --- | --- | --- |
| EGFP | Lifeact-EGFP | WT F-Tractin | F-tractin_opt_ | R19A F-tractin_opt_ | F29A F-tractin_opt_ |
| 2.31991 | 0.9515 | 1.02518 | 0.98251 | 1.8729 | 1.96703 |
| 2.11156 | 0.91524 | 0.81472 | 1.05411 | 1.71824 | 1.9501 |
| 1.70054 | 0.91314 | 0.85063 | 1.32503 | 1.3185 | 2.59131 |
| 1.91516 | 1.06072 | 1.12005 | 1.32822 | 1.43325 | 2.47616 |
| 1.82647 | 0.74157 | 1.16625 | 1.08952 | 1.17977 | 2.32414 |
| 2.61161 | 1.278 | 0.42938 | 1.26378 | 1.86086 | 1.96269 |
| 2.25339 | 0.23638 | 1.83873 | 1.24959 | 1.3022 | 1.91924 |
| 3.0425 | 0.77384 | 1.07703 | 0.8488 | 1.22539 | 2.48995 |
| 2.24787 | 0.92266 | 1.03179 | 0.82999 | 1.50241 | 1.90708 |
| 1.60767 | 1.5496 | 0.93294 | 1.24371 | 1.12466 | 2.29064 |
| 1.51929 | 0.9234 | 0.79192 | 1.39514 | 1.95383 | 2.08093 |
| 2.28148 | 1.17317 | 0.8471 | 1.0572 | 1.39285 | 0.72076 |
| 1.82058 | 0.66659 | 1.05184 | 0.81586 | 1.28641 | 1.99262 |
| 2.45656 | 1.15827 | 1.00463 | 0.94588 | 1.32429 | 2.08492 |
| 2.65255 | 0.8547 | 0.88356 | 1.03255 | 1.42545 | 1.47013 |
| 2.30843 | 1.14019 | 0.92152 | 1.16902 | 1.54786 | 1.41062 |
| 2.32307 | 0.69451 | 0.75439 | 1.04272 | 1.43033 | 1.97664 |
| 2.40117 | 0.85937 | 0.86335 | 1.01187 | 1.79863 | 2.02028 |
| 1.96718 | 1.10593 | 1.04163 | 1.14848 | 2.16267 | 1.30012 |
| 0.4874 | 0.6289 | 1.21876 | 0.96642 | 1.20876 | 1.78288 |
| 1.86853 | 0.96511 | 0.69085 | 1.09544 | 1.82378 | 2.07646 |
| 1.97962 | 1.20564 | 0.78758 | 1.42807 | 1.31567 | 2.67368 |
| 0.56688 | 0.6776 | 1.16551 | 1.10389 | 1.71339 | 1.86166 |
| 2.85468 | 1.3421 | 0.96267 | 1.17929 | 1.51893 | 2.07962 |
| 2.5068 | 1.06469 | 0.84917 | 1.37009 | 2.34058 | 1.85723 |
| 2.13662 | 1.05086 | 0.91831 | 0.90726 | 1.66073 | 1.80141 |
| 2.66455 | 0.90025 | 1.01507 | 1.06158 | 0.79781 | 1.7028 |
| 0.8389 | 0.91566 | 1.00948 | 1.02026 | 1.39963 | 2.05217 |
| 2.99019 | 1.30739 | 0.94784 | 1.12912 | 1.41221 | 1.53976 |
| 1.40293 | 0.8434 | 0.9957 | 1.13508 | 1.17617 |  |
| 3.1199 | 1.09165 | 1.13471 | 1.1771 | 1.13419 |  |
| 2.60425 | 1.12198 | 1.06141 | 1.07488 |  |  |
| 2.66563 | 1.11298 | 1.53377 |  |  |  |
| 1.75229 | 1.3455 | 1.4993 |  |  |  |
| 2.24508 | 0.94285 | 0.91536 |  |  |  |
| 0.4749 | 0.7925 | 0.94995 |  |  |  |
| 2.90781 | 0.93688 | 1.00336 |  |  |  |
| 1.69304 | 0.71965 | 0.92489 |  |  |  |
| 2.71161 | 1.1586 | 1.23807 |  |  |  |
| 2.71384 | 1.10301 | 0.99592 |  |  |  |
| 2.63481 | 0.83908 | 1.06001 |  |  |  |
| 2.41208 | 1.00892 | 1.09331 |  |  |  |
| 2.69272 | 1.03454 | 1.24456 |  |  |  |
| 2.35117 | 0.97103 | 1.08788 |  |  |  |
| 2.61031 | 0.79321 | 1.11168 |  |  |  |
| 2.48106 | 0.66786 | 0.85232 |  |  |  |
| 2.75409 | 0.65243 | 1.09737 |  |  |  |
|  | 0.64238 | 1.02868 |  |  |  |
|  | 0.76136 | 1.25835 |  |  |  |
|  | 1.28458 | 0.90932 |  |  |  |

**Figure 3 H**

| Half time recovery – For each photobleached ROI | | | | | |
| --- | --- | --- | --- | --- | --- |
| EGFP | Lifeact | WT-tractin | F-tractin_opt_ | F29A F-tractin_opt_ | R19A F-tractin_opt_ |
| 0.09 | 0.28 | 0.55 | 0.74 | 0.13 | 0.5 |
| 0.19 | 0.3 | 0.51 | 0.7 | 0.12 | 0.32 |
| 0.22 | 0.37 | 0.47 | 0.61 | 0.22 | 0.23 |
| 0.29 | 0.29 | 0.3 | 0.68 | 0.23 | 0.31 |
| 0.06 | 0.19 | 0.43 | 0.24 | 0.19 | 0.27 |
| 0.16 | 0.18 | 0.76 | 0.47 | 0.62 | 0.17 |
| 0.11 | 0.52 | 0.8 | 0.67 | 0.12 | 0.27 |
| 0.28 | 1.2 | 0.71 | 0.56 | 1.25 | 0.33 |
| 0.04 | 0.44 | 0.9 | 0.41 | 0.24 | 0.25 |
| 0.15 | 0.34 | 0.53 | 0.73 | 0.2 | 0.21 |
| 0.17 | 0.26 | 0.77 | 0.38 | 1.19 | 0.21 |
| 0.22 | 0.52 | 0.46 | 0.34 | 0.48 | 0.28 |
| 0.25 | 0.27 | 0.86 | 0.47 | 0.17 | 0.29 |
| 0.23 | 0.26 | 0.68 | 0.31 | 0.17 | 0.54 |
| 0.44 | 0.44 | 0.9 | 0.29 | 0.43 | 0.28 |
| 0.15 | 0.52 | 0.77 | 1.26 | 0.2 | 0.31 |
| 0.07 | 0.62 | 0.98 | 0.35 | 0.16 | 0.29 |
| 0.12 | 0.41 | 0.61 | 0.64 | 0.31 | 0.39 |
| 0.19 | 0.4 | 0.42 | 0.35 | 0.17 | 0.19 |
| 0.14 | 0.8 | 0.46 | 0.35 | 0.16 | 0.25 |
| 0.05 | 0.26 | 0.91 | 0.61 | 0.14 | 0.32 |
| 0.16 | 0.16 | 0.74 | 0.54 | 0.11 | 0.2 |
| 0.14 | 0.44 | 0.96 | 0.57 | 0.28 | 0.53 |
| 0.14 | 0.53 | 0.39 | 0.52 | 0.54 | 0.33 |
| 0.12 | 0.57 | 0.48 | 0.22 | 0.11 | 0.17 |
| 0.16 | 0.42 | 0.35 | 0.3 | 0.06 | 0.39 |
| 0.26 | 0.48 | 0.76 | 0.41 | 0.15 | 0.43 |
| 0.2 | 0.37 | 0.49 | 0.29 | 1.11 | 0.86 |
| 0.4 | 0.22 | 0.37 | 0.27 | 0.09 | 0.24 |
| 0.17 | 0.25 | 1.04 | 0.65 | 0.19 | 0.25 |
| 0.13 | 0.33 | 2.85 | 0.43 | 0.31 | 0.59 |
| 0.14 | 0.35 | 0.59 | 0.74 | 0.31 | 0.25 |
| 0.25 | 0.48 | 1.27 | 0.3 | 0.5 | 0.18 |
| 0.13 | 0.27 | 0.75 | 0.57 | 0.13 | 0.31 |
| 0.31 | 0.26 | 0.88 | 0.4 | 0.21 | 0.3 |
| 0.57 | 0.35 | 0.51 | 0.41 | 0.39 | 0.29 |
| 0.08 | 0.27 | 0.74 | 0.65 | 0.13 | 0.55 |
| 0.04 | 0.31 | 0.69 | 0.43 | 0.25 | 0.36 |
| 0.27 | 0.52 | 0.66 | 0.49 | 0.26 | 0.22 |
| 0.4 | 0.77 | 0.67 | 0.57 | 0.3 | 0.24 |
| 0.22 | 0.48 | 0.67 | 0.69 | 0.18 | 1.23 |
| 0.18 | 0.16 | 0.32 | 0.54 | 0.3 | 0.38 |
| 0.3 | 0.57 | 0.4 | 1 | 0.31 | 0.31 |
| 0.41 | 0.32 | 0.71 | 0.47 | 0.47 | 0.34 |
| 0.19 | 0.5 | 1.17 | 0.28 | 0.19 | 0.24 |
| 0.43 | 0.85 | 1.7 | 0.47 | 0.76 | 0.25 |
| 0.52 | 0.32 | 0.58 | 0.42 | 0.41 | 0.23 |
| 0.27 | 0.44 | 0.48 | 0.43 | 0.46 | 0.37 |
| 0.27 | 0.44 | 0.22 | 1.26 | 0.31 | 0.55 |
| 0.3 | 0.48 | 0.6 | 0.34 | 0.16 | 0.62 |
| 0.48 | 0.31 | 0.73 | 1.01 | 2.72 | 0.64 |
| 0.38 | 0.42 | 0.48 | 0.86 | 0.46 | 0.49 |
| 0.34 | 0.36 | 0.56 | 0.33 | 0.23 | 0.19 |
| 0.17 | 0.29 | 0.78 | 0.19 | 0.24 | 0.31 |
| 0.16 | 0.39 | 0.42 | 0.48 | 0.42 | 0.27 |
| 0.43 | 0.45 | 0.53 | 0.45 | 0.7 | 0.46 |
| 0.41 | 0.69 | 1.46 | 0.6 | 0.16 | 0.26 |
| 0.31 | 0.36 | 0.41 | 0.35 | 0.26 | 0.34 |
| 0.25 | 0.57 | 0.45 | 0.56 | 0.31 | 0.36 |
| 0.26 | 0.69 | 0.75 | 0.24 | 0.44 | 0.43 |
| 0.27 | 0.12 | 0.57 | 0.71 | 0.22 | 0.37 |
| 0.8 | 0.45 | 0.84 | 0.68 | 0.12 | 0.31 |
| 2.01 | 0.19 | 0.91 | 0.8 | 0.28 | 0.41 |
| 0.4 | 0.61 | 0.59 | 0.48 | 0.42 | 0.41 |
| 0.14 | 0.3 | 0.46 | 1 | 0.23 | 0.21 |
| 0.23 | 0.43 | 1.04 | 1.06 | 0.13 | 0.55 |
| 0.34 | 0.91 | 0.38 | 0.42 | 0.45 | 0.69 |
| 0.35 | 0.4 | 0.53 | 0.55 | 1.01 | 0.38 |
| 0.32 | 0.35 | 0.8 | 0.56 | 0.21 | 0.2 |
| 0.26 | 0.4 | 0.51 | 0.18 | 0.27 | 0.3 |
| 0.23 | 0.85 | 0.69 | 0.49 | 0.22 | 0.24 |
| 0.73 | 0.95 | 0.63 | 0.4 | 0.33 | 0.6 |
| 0.26 | 0.55 | 0.83 | 0.62 | 0.12 | 0.26 |
| 0.28 | 0.27 | 0.94 | 0.31 | 0.27 | 0.33 |
| 0.21 | 0.15 | 0.75 | 0.27 | 0.3 | 0.23 |
| 0.5 | 0.24 | 1.93 | 0.37 | 0.39 | 0.24 |
| 1.84 | 0.4 | 0.29 | 0.19 | 0.23 | 0.46 |
| 0.17 | 0.4 | 0.44 | 0.19 | 0.15 | 0.37 |
| 0.58 | 0.48 | 0.66 | 0.2 | 0.2 | 0.11 |
| 0.31 | 0.44 | 0.39 | 0.38 | 0.26 | 0.47 |
| 0.23 | 0.43 | 1.37 | 0.18 | 0.3 | 0.3 |
| 0.52 | 0.68 | 0.4 | 0.65 | 0.34 | 0.46 |
| 0.41 | 0.5 | 0.43 | 0.35 | 0.14 | 0.17 |
| 0.64 | 0.3 | 0.55 | 0.57 | 0 | 0.78 |
| 0.23 | 1.56 | 0.71 | 0.43 | 0.24 | 0.19 |
| 0.32 | 0.69 | 0.81 | 0.53 | 0.23 | 0.24 |
| 0.24 | 7.88 | 0.72 | 0.44 | 0.43 | 0.25 |
| 0.42 | 0.49 | 0.29 | 0.25 | 0.11 | 0.4 |
| 0.35 | 0.14 | 0.99 | 0.64 | 0.25 | 0.37 |
| 0.28 | 0.58 | 0.46 | 0.54 | 0.64 | 0.4 |
| 0.28 | 0.47 | 0.77 | 0.53 | 0.28 | 0.22 |
| 0.19 | 0.38 | 0.3 | 0.52 | 0.33 | 0.72 |
| 0.36 | 0.41 | 0.68 | 0.29 | 0.24 | 0.35 |
| 0.45 | 0.39 | 1.08 | 0.96 | 0.18 | 0.42 |
| 0.25 | 0.41 | 0.62 | 0.78 | 0.36 | 0.66 |
| 0.39 | 0.48 | 0.64 | 0.77 | 0.21 | 0.53 |
| 0.33 | 0.99 | 0.55 | 0.28 | 0.12 | 0.37 |
| 0.45 | 0.46 | 0.42 | 0.54 | 5.45 | 0.36 |
| 0.23 | 0.5 | 0.85 | 0.61 | 0.22 | 0.58 |
| 0.27 | 0.49 | 0.43 | 0.89 | 0.16 | 0.09 |
| 0.16 | 0.43 | 0.32 | 0.51 | 0.4 | 1.03 |
| 0.24 | 0.33 | 0.45 | 0.34 | 0.32 | 0.42 |
| 0.29 | 0.3 | 0.53 | 0.34 | 0.3 | 0.43 |
| 0.42 | 0.46 | 0.54 | 0.3 | 0.24 | 0.32 |
| 0.18 | 2.96 | 0.44 | 0.48 | 0.21 | 0.25 |
| 0.22 | 0.14 | 0.77 | 0.67 | 0.16 | 0.41 |
| 0.35 | 0.72 | 0.57 | 0.63 | 0.12 | 0.81 |
| 0.39 | 0.45 | 0.81 | 0.61 | 0.35 | 0.79 |
| 0.42 | 0.36 | 0.7 | 0.75 |  | 0.4 |
| 0.23 | 0.23 | 0.48 | 0.32 |  | 0.28 |
| 0.33 | 1.42 | 0.39 | 0.51 |  | 0.29 |
| 0.28 | 0.61 | 0.59 | 0.67 |  | 0.44 |
| 0.29 | 0.28 |  | 0.88 |  | 0.35 |
| 0.39 | 0.54 |  | 0.79 |  | 0.25 |
| 0.28 | 0.38 |  | 0.49 |  | 0.31 |
| 0.28 | 0.2 |  | 0.48 |  | 0.46 |
| 0.33 | 1.26 |  | 1.95 |  | 0.31 |
| 0.48 | 0.37 |  | 0.56 |  | 0.35 |
| 0.25 | 0.41 |  | 0.62 |  | 0.51 |
| 0.13 |  |  | 0.68 |  | 0.52 |
| 0.21 |  |  |  |  | 0.29 |
| 0.25 |  |  |  |  | 0.19 |
| 0.26 |  |  |  |  | 0.51 |
| 0.37 |  |  |  |  | 0.26 |
| 0.21 |  |  |  |  | 0.33 |
| 0.21 |  |  |  |  | 1.38 |
| 0.31 |  |  |  |  | 0.36 |
| 0.31 |  |  |  |  | 0.5 |
| 0.51 |  |  |  |  | 0.72 |
| 0.22 |  |  |  |  | 0.39 |
| 0.35 |  |  |  |  | 0.34 |
| 0.18 |  |  |  |  | 1.99 |
| 0.29 |  |  |  |  |  |
| 0.26 |  |  |  |  |  |

**Figure 4 F**

|  |  | ADP-ribosylation level | | |
| --- | --- | --- | --- | --- |
|  | Peptide, µM | Repeat 1 | Repeat 2 | Repeat 3 |
|  | 0 | 1 | 1 | 1 |
| F-tractin_opt_ | 1 | 1,245716 | 0,95033 | 1,419998 |
|  | 3 | 1,210256 | 1,296322 | 1,29383 |
|  | 10 | 0,846657 | 0,728925 | 1,078508 |
|  | 30 | 0,320904 | 0,220558 | 0,140877 |
|  | 100 | 0,032604 | 0,100767 | 0,002262 |
| F29A F-tractin_opt_ | 100 | 0,715886 | 1,144903 | 1,162086 |
| Lifeact | 1 | 0,978733 | 0,811616 | 1,268379 |
|  | 3 | 0,825802 | 0,671345 | 1,323177 |
|  | 10 | 0,671724 | 1,132312 | 0,970817 |
|  | 30 | 0,313245 | 0,438167 | 0,331448 |
|  | 100 | 0 | 0,032044 | 0,093063 |
