# Supplementary material for: Structure of the F-tractin–F-actin complex
Source: J Cell Biol. 2025 Feb 10;224(4):e202409192. doi: 10.1083/jcb.202409192 (PMC11809415; doi:10.1083/jcb.202409192)

Figure 2C, top panel

Low-speed sedimentation, 10,000 g

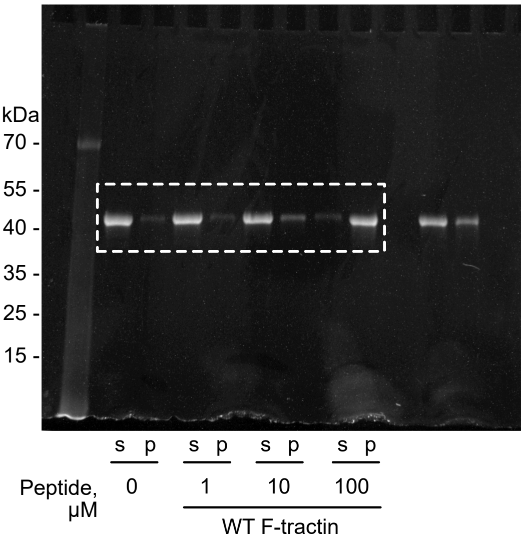

Figure 2C, bottom panel

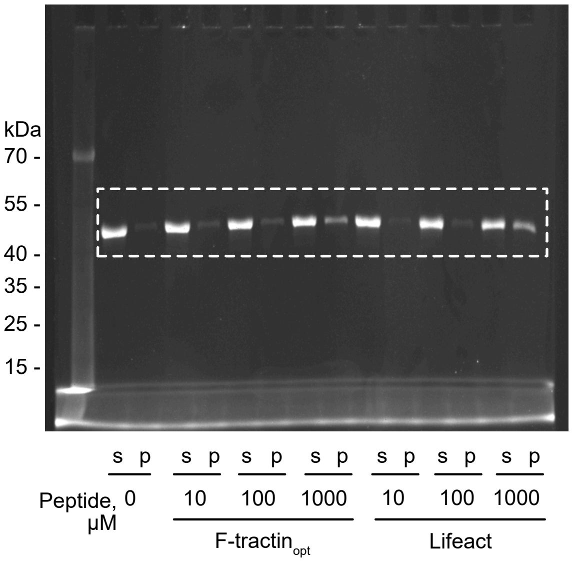

Figure 2F, F-tractin$_{opt}$

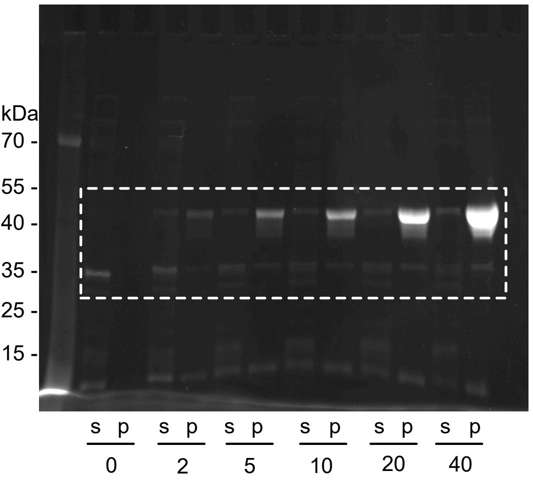

Figure 2F, F29A F-tractin$_{opt}$

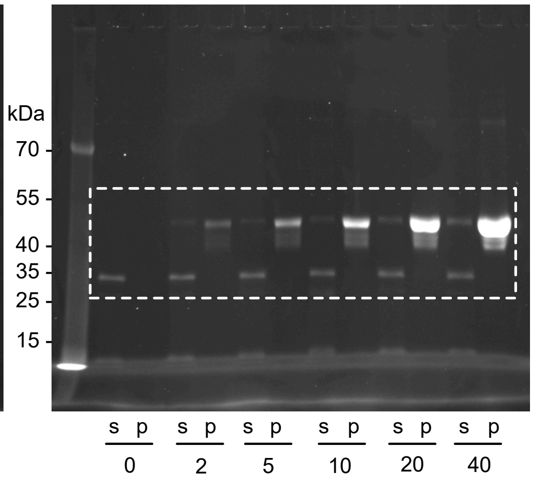

Figure 2F, R19A F-tractin$_{opt}$

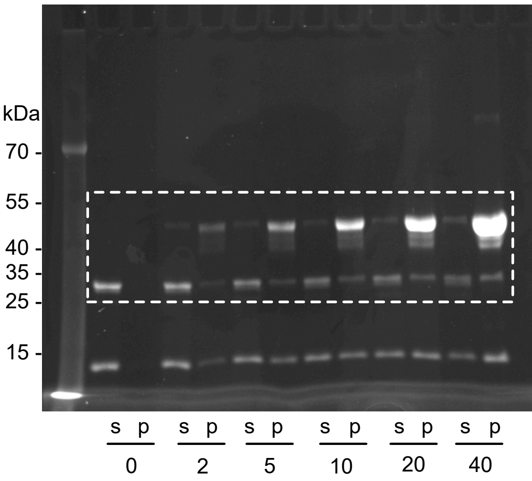

Supplement: SourceData F2 — is the source file for Fig. 2. [file jcb_202409192_sourcedataf2.pdf]
