# Supplementary figures and images for: Structure of the F-tractin–F-actin complex
Source: J Cell Biol. 2025 Feb 10;224(4):e202409192. doi: 10.1083/jcb.202409192 (PMC11809415; doi:10.1083/jcb.202409192)

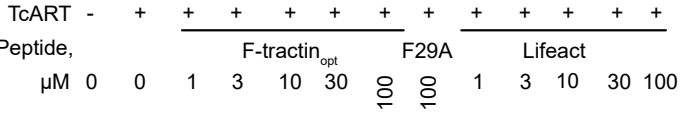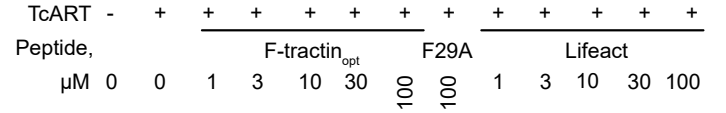

Supplement: SourceData F4 — is the source file for Fig. 4. [file jcb_202409192_sourcedataf4.pdf]
